# Socioeconomic Status and Subclinical Coronary Disease in the Whitehall II Epidemiological Study

**DOI:** 10.1371/journal.pone.0008874

**Published:** 2010-01-25

**Authors:** Andrew Steptoe, Mark Hamer, Katie O'Donnell, Shreenidhi Venuraju, Michael G. Marmot, Avijit Lahiri

**Affiliations:** 1 Department of Epidemiology and Public Health, University College London, London, United Kingdom; 2 Cardiac Imaging and Research Centre, Wellington Hospital, London, United Kingdom; New York University School of Medicine, United States of America

## Abstract

**Background:**

There are pronounced socioeconomic disparities in coronary heart disease, but the extent to which these primarily reflect gradients in underlying coronary artery disease severity or in the clinical manifestation of advanced disease is uncertain. We measured the relationship between socioeconomic status (SES) as indexed by grade of employment and coronary artery calcification (CAC) in the Whitehall II epidemiological cohort, and tested the contribution of lifestyle, biological and psychosocial factors in accounting for this association.

**Methods and Findings:**

CAC was assessed in 528 asymptomatic men and women aged 53–76 years, stratified into higher, intermediate and lower by grade of employment groups. Lifestyle (smoking, body mass index, alcohol consumption, physical activity), biological (blood pressure, lipids, fasting glucose, inflammatory markers) and psychosocial factors (work stress, financial strain, social support, depression, hostility, optimism) were also measured. Detectable CAC was present in 293 participants (55.5%). The presence of calcification was related to lifestyle and biological risk factors, but not to grade of employment. But among individuals with detectable calcification, the severity of CAC was inversely associated with grade of employment (p = 0.010), and this relationship remained after controlling for demographic, lifestyle, biological and psychosocial factors. Compared with the higher grade group, there was a mean increase in log Agatston scores of 0.783 (95% C.I. 0.265–1.302, p = 0.003) in the intermediate and 0.941 (C.I. 0.226–1.657, p = 0.010) in the lower grade of employment groups, after adjustment for demographic, lifestyle, biological and psychosocial factors.

**Conclusions:**

Low grade of employment did not predict the presence of calcification in this cohort, but was related to the severity of CAC. These findings suggest that lower SES may be particularly relevant at advanced stages of subclinical coronary artery disease, when calcification has developed.

## Introduction

There is a pronounced socioeconomic gradient in coronary heart disease, with greater morbidity and mortality among people of lower socioeconomic status (SES) as defined by occupational position, education and income [1], [2]. The extent to which this pattern reflects SES gradients in underlying coronary artery disease severity or in the clinical manifestation of advanced coronary disease is uncertain. SES disparities in the triggering of myocardial infarction and in sudden cardiac death have been observed [3], [4], and variations in delay in seeking medical care following the onset of acute coronary syndromes may exacerbate morbidity [5]. Low SES is also associated with greater short-term fatality following acute coronary syndrome and coronary artery bypass graft [6]. Carotid plaque and intimal-medial thickness (IMT), surrogate markers of coronary atherosclerotic burden, are related to SES both cross-sectionally and longitudinally [7]–[9], but evaluations of subclinical coronary disease are limited and findings have been less consistent, and largely attributable to biological risk factors [10]–[12].

This study investigated the relationship between SES and coronary artery calcification (CAC) in a well-characterized healthy older population cohort in which associations between SES as defined by grade of employment, cardiovascular risk factors, coronary heart disease incidence and psychosocial factors have already been established [13]–[16]. We analyzed both the presence versus absence of detectable CAC, and associations with extent of CAC in participants with any calcification. We also tested the role of lifestyle (smoking, alcohol consumption, physical activity), biological (blood pressure, lipid profile, fasting glucose, inflammatory markers) and psychosocial factors (work stress, financial strain, social networks, social support, depressed mood, hostility, optimism) in accounting for grade of emplyment differences in coronary calcification.

## Materials and Methods

### Participants

All participants gave full informed consent, and ethical approval was obtained from the UCLH Committee on the Ethics of Human Research. The Heart Scan study involved a sub-sample of participants in the Whitehall II cohort, recruited for the assessment of CAC in 2006 to 2008. The Whitehall II epidemiological cohort is a sample of 10,308 London-based civil servants recruited in 1985–1988 when aged 35–55 years to investigate demographic, psychosocial and biological risk factors for coronary heart disease [13]. Participants in this sub-sample were of white European origin, had no history or objective signs of coronary heart disease, no previous diagnosis or treatment for hypertension, diabetes, inflammatory diseases, or allergies. Socioeconomic status was defined by grade of employment within the British civil service, and recruitment was stratified to ensure adequate representation of higher, intermediate and lower grade of employment groups. A total of 1169 participants in Whitehall II were approached, but 27.6% were not eligible (mainly because of prescribed medications) and 25.9% declined to take part. The total sample of 543 included 294 men and 249 women aged 53–76 years. Fifteen individuals had missing data on one or more of the factors included in these analyses, leaving a final sample of 528.

### Measurement of Socioeconomic Status

Grade of employment was used as the indicator of SES, since this has previously been shown both in the original Whitehall and Whitehall II studies to predict cardiovascular risk and mortality [13], [16], [17]. Participants were classified on their current (if still employed) or most recent civil service grade into higher, intermediate and lower status groups. The 12 possible employment grades of the civil service were classified into the three categories analyzed in this study as follows: administrative assistant, administrative officer and executive office (lower), higher executive office and senior executive office (intermediate), grades 7 to 1 (higher). Grade of employment was strongly associated with other commonly used markers of SES such as educational attainment, personal and household income.

### Measurement of Subclinical Coronary Disease

Coronary artery calcification was performed using electron beam computed tomography (GE Imatron C-150, San Francisco, CA) as described elsewhere [18]. In brief, 40 contiguous 3-mm slices were obtained during a single breath-hold starting at the carina and proceeding to the level of the diaphragm. Scan time was 100 ms/slice, synchronized to 40% of the R-R interval. Agatston scores were calculated to quantify the extent of CAC by a single experienced investigator blinded to the clinical data on an Aquarius workstation (TeraRecon Inc., San Mateo, CA).

### Biological and Anthropometric Measures

Height and weight were measured by an experienced nurse, from which body mass index (BMI) was computed. Blood pressure was measured while seated using a digital monitor. A fasting blood sample was drawn for the analysis of total cholesterol, high density lipoprotein (HDL) cholesterol, plasma triglycerides, fasting glucose, high sensitivity C-reactive protein and interleukin (IL) 6, using methods described previously [13], [16].

### Lifestyle and Psychosocial Measures

Participants reported whether or not they were currently in paid employment, current smoking status, and weekly alcohol intake by questionnaire. Physical exercise was assessed by enquiring about the frequency of moderate and vigorous activities, and responses were classified into three categories: none, up to twice per week, and three times per week or more. Work stress was assessed in terms of job demands and job control, and financial strain, social support, social networks, depression, hostility and optimism were measured using standard questionnaires [14], [19]–[22]. The Cronbach α scores for the psychosocial measures ranged from 0.73 to 0.94.

### Statistical Analysis

The associations between grade of employment and lifestyle, biological and psychosocial factors were analyzed using analysis of variance for continuous and chi-squared statistics for categorical variables respectively. Detectable CAC was recorded in 293 (55.5%) of participants. The distribution of CAC scores violated the statistical assumptions underlying linear modeling even after log transformation, making them unsuitable for linear regression analysis of the complete sample. Associations between CAC and grade of employment were therefore analyzed in two ways. Firstly, logistic regression was used to investigate associations with the presence vs absence of CAC. Socioeconomic status was modeled as a categorical variable based on the three grade of employment groups. Second, the CAC scores of individuals with detectable calcification were log transformed, and were subsequently normally distributed. Analysis of covariance was therefore carried out on log transformed CAC scores with grade of employment as a between-person factor. Four models were tested: model 1 included demographic factors (gender, age, employment status, and statin use) as covariates. Lifestyle factors (BMI, smoking, alcohol consumption, and physical activity) were added in model 2, biological risk factors (systolic blood pressure, fasting total cholesterol, HDL-cholesterol, triglycerides, fasting glucose, C-reactive protein and IL-6) in model 3, and psychosocial factors (financial strain, social network size, social support, depressed mood, hostility, and optimism) in model 4. None of the variables included in these models showed multicollinearity according to variance inflation factor and tolerance values.

## Results

There was no difference in the proportion of men and women in the three grade of employment groups, but lower grade participants were an average 2 years older than those in higher and intermediate grade groups (p = 0.004, see Table 1). The proportion of smokers in the sample was low, and did not vary with grade of employment. Forty-five participants were taking statins, but this was not related to grade of employment. The higher grade group consumed more alcohol (p<0.001). Lower grade of employment was associated with higher systolic BP and lower HDL-cholesterol, but other biological factors did not differ across groups. Financial strain was generally low, but was inversely correlated with grade of employment (p<0.001). Lower grade of employment was also associated with less job control (p<0.001), smaller social networks (p = 0.003), greater hostility (p = 0.002) and less optimism (p = 0.004), but also with greater job demands (p<0.001).

**Table 1:** Grade of employment and risk factors.

	Higher grade of employment (n = 199)	Intermediate grade of employment (n = 209)	Lower grade of employment (n = 120)	P^1^
Men/women	108/91	124/85	51/69	0.094
Age (years)	62.3 (5.5)	62.6 (5.4)	64.4 (6.0)	0.005
Paid employment (%)	85 (42.7%)	72 (34.4%)	41 (34.2%)	0.79
Current smokers (%)	10 (5.0%)	12 (5.7%)	8 (6.7%)	0.31
Body mass index(kg/m^2^)	25.7 (0.28)	25.9 (0.27)	26.1 (0.36)	0.42
Alcohol intake (units/w)	11.31 (0.62)	8.13 (0.60)	7.11 (0.80)	0.001
Moderate/vigorous physical activity (%):				
None	21 (10.6%)	22 (10.5%)	20 (16.7%)	0.15
Up to 2/week	118 (59.3%)	126 (60.3%)	72 (60.0%)	
3/week or more	60 (30.2%)	61 (29.2%)	28 (23.3%)	
Systolic blood pressure (mmHg)	122.3 (1.13)	123.9 (1.09)	126.4 (1.45)	0.025
Total cholesterol (mmol/l)	5.95 (0.07)	5.81 (0.07)	5.79 (0.09)	0.15
HDL-cholesterol (mmol/l)	1.78 (0.03)	1.67 (0.03)	1.62(0.04)	0.002
Triglycerides (mmol/l)	1.21 (0.06)	1.20 (0.05)	1.36 (0.71)	0.10
Fasting glucose (mmol/l)	5.19 (0.04)	5.18 (0.04)	5.18 (0.05)	0.88
C-reactive protein (µg/ml)	1.68 (0.15)	1.58 (0.15)	1.72 (0.20)	0.87
IL-6 (pg/ml)	1.78 (0.09)	1.76 (0.09)	1.92 (0.12)	0.36
Job demands (%)	66.8 (1.35)	58.5 (1.32)	47.8 (1.75)	0.001
Job control (%)	68.7 (1.22)	59.5 (1.19)	51.4 (1.59)	0.001
Financial strain (%)	4.55 (1.23)	12.54 (1.19)	14.15 (1.60)	0.001
Social network (0–11)	4.49 (0.11)	4.04 (0.11)	3.94 (0.15)	0.003
Social support (%)	75.37 (1.74)	72.06 (1.68)	69.98 (2.26)	0.060
Depressed mood (CESD) (0–60)	6.09 (0.47)	6.98 (0.45)	7.02 (0.61)	0.23
Hostility (0–10)	21.83 (1.67)	27.71 (1.63)	30.36 (2.20)	0.002
Optimism (%)	68.50 (1.19)	64.26 (1.15)	62.88 (1.55)	0.004

Mean (s.e.m.) and N (%).

1 *P* for trend across grade of employment groups, adjusted for gender and age.

HDL = high density lipoprotein, IL-6 = interleukin 6. CESD = Center for Epidemiologic Studies Depression scale.

### Grade of Employment and Coronary Artery Calcification

CAC was present in 293 (55.5% of participants), with 166 (31.4%) having Agatston scores <100, 76 (14.4%) between 100 and 399, and 51 (9.7%) scores of 400 or over. Detectable calcification was positively related to age (p<0.001), and was more common among men than women (68.1% vs 41.0%), a difference that remained significant after adjustment for age (p<0.001). The associations between presence of CAC and risk factors are summarized in Table 2. CAC was more likely to be present among smokers (p = 0.041), individuals with greater BMI (p = 0.077), and unexpectedly with increased regular physical activity (p = 0.006), after adjustment for age and gender. The presence of CAC was also positively associated with systolic blood pressure (p = 0.032), and total cholesterol (p = 0.034). It was not related to other metabolic or to inflammatory markers. Nor was it associated either with grade of employment or psychosocial factors. Overall, CAC was present in 55.0% (95% C.I. 48.1–61.9) of higher, 61.2% (C.I. 54.5–68.0) of intermediate, and 46.2% (C.I. 37.3–55.1) of lower grade of employment participants. Separate analysis of men and women produced similar results.

**Table 2:** Factors associated with the presence of coronary artery calcification.

Factor	Comparison unit	Odds of CAC adjusted for age and gender (95% C.I.)	*P*
Current smoker	Non-smokers	2.389 (1.035 to 5.517)	0.041
Body mass index	Unit increase	1.044 (0.995 to 1.095)	0.077
Alcohol intake	Unit increase	1.011 (0.989 to 1.033)	0.34
Moderate/vigorous physical activity	Level increase	1.543 (1.131 to 2.105)	0.006
Systolic blood pressure	1 mm increase	1.013 (1.001 to 1.025)	0.032
Total cholesterol	1 mmol/l increase	1.226 (1.015 to 1.481)	0.034
HDL-cholesterol	1 mmol/l increase	0.947 (0.613 to 1.463)	0.81
Triglycerides	1 mmol/l increase	1.122 (0.872 to 1.443)	0.37
Fasting glucose	1 mmol/l increase	1.031 (0.709 to 1.500)	0.87
C-reactive protein	1 µg/ml increase	0.977 (0.895 to 1.066)	0.60
IL-6	1 pg/ml increase	1.014 (0.879 to 1.171)	0.85
Grade of employment	Reduced grade level	0.831 (0.651 to 1.061)	0.14

**Figure 1:**
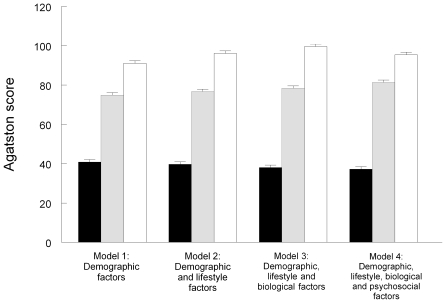
Geometric means for Agatston scores in the higher (solid bars), intermediate (striped bars) and lower (open bars) grade of employment groups (n = 293). Error bars are standard errors of the mean. Model 1 is adjusted for age, gender, employment status, and statin use. Model 2 is additionally adjusted for BMI, smoking, alcohol consumption and physical activity. Model 3 is additionally adjusted for systolic blood pressure, total and HDL-cholesterol, triglycerides, fasting glucose, IL-6 and C-reactive protein. Model 4 is additionally adjusted for financial strain, social network size, social support, job demands, job control, depressed mood, hostility, and optimism.

The Agatston scores of the 293 participants with detectable CAC were positively related to age (p<0.001), and were greater in men than women (p = 0.011) and in statin users (p<0.001). Agatston scores also showed a significant association with grade of employment (p = 0.010), with greater CAC in lower grade men and women after adjusting for age, gender, employment status, and statin use (Table 3). Inclusion of lifestyle factors, biological and psychosocial factors did not alter the inverse association appreciably (p = 0.010). These effects are illustrated in Figure 1; Agatston scores were more than twice as great in the lower than in the higher grade of employment groups in all models. The association was unchanged with the inclusion of waist/hip ratio and glycated haemoglobin as covariates in a reduced sample (results not shown).

**Table 3:** Grade of employment and the extent of coronary artery calcification.

Grade of employment group	Agatston score (Log)	Mean difference (95% C.I.) adjusted for demographic factors^1^		Mean difference (95% C.I.) adjusted for demographic and lifestyle factors^2^		Mean difference (95% C.I.) adjusted for demographic, lifestyle and biological factors^3^		Mean difference (95% C.I.) adjusted for demographic, lifestyle, biological and psychosocial factors^4^	
	**Mean ± S.E.M.**		P		P		P		P
Higher	3.75±1.84	Reference		Reference		Reference		Reference	
Intermediate	4.25±1.83	+0.596 (0.140 to 1.052)	0.011	+0.660 (0.197 to 1.123)	0.005	+0.722 (0.255 to 1.189)	0.004	+0.783 (0.265 to 1.302)	0.003
Lower	4.64±1.92	+0.824 (0.242 to 1.405)	0.006	+0.884 (0.296 to 1.472)	0.003	+0.961 (0.358 to 1.565)	0.002	+0.941 (0.226 to 1.657)	0.010

Analyses of 293 participants with detectable coronary calcification.

1 Model 1 adjusted for age, gender, employment status, and statin use.

2 Model 2 as for Model 1, plus BMI, smoking, alcohol consumption and physical activity.

3 Model 3, as for Model 2, plus systolic blood pressure, total and HDL-cholesterol, triglycerides, fasting glucose, IL-6 and C-reactive protein.

4 Model 4, as for Model 3, plus financial strain, social network size, social support, job demands, job control, depressed mood, hostility, and optimism.

## Discussion

This study investigated the association between SES defined by grade of employment and subclinical coronary artery disease as indexed by CAC, in a well-characterized sample of healthy older men and women. The presence of CAC was more common with advancing age, and was related to gender, biological and lifestyle risk factors. It was not, however, associated with grade of employment. But among participants with detectable CAC, Agatston scores were inversely related to grade of employment. A social gradient was present, with the greatest CAC in the lowest grade of employment group, and moderate CAC in the intermediate grade participants. These disparities were largely unaffected by controlling statistically for lifestyle, biological and psychosocial risk factors.

Previous studies of SES differences in CAC have been inconsistent. Colhoun et al [23] reported that CAC was more common in blue than white collar men and women, but effects were no longer significant after age, gender and risk factors had been taken into account. An analysis of the Multi-Ethnic Study of Atherosclerosis (MESA) demonstrated an inverse association between education and the presence of CAC in white participants but not in other ethnic groups [11]; the difference was no longer significant after adjustment for smoking, BMI, lipid levels, hypertension and diabetes. By contrast, Yan et al [24] showed that the prevalence of CAC was inversely associated with education in the Coronary Artery Risk Development in Young Adults (CARDIA) study, and the difference persisted after changes in risk factors over the previous 15 years had been taken into account. The study involved a young cohort (age 33–45 years) in which the prevalence of CAC was low (9.3%). An inverse association between CAC and SES defined by education or income has been reported in a large sample of German men and women, where differences were largely accounted for by standard cardiovascular risk factors [10]. Other studies have failed to document differences in the presence of CAC by SES defined by income, education or occupational grade in asymptomatic individuals [12], [25].

There is consistent evidence that SES is associated with carotid IMT and plaque independent of risk factors [7]–[9]. But more direct assessments of the coronary arteries with EBCT and dual source CT scans cast doubt on the impact of SES on the early manifestations of coronary artery disease. The robust relationship that we observed between lower grade of employment and the severity of CAC among individuals with detectable calcification suggests that the impact of SES may be apparent at more advanced stages of subclinical coronary artery disease. Alternatively, grade of employment may influence the progression of CAC among individuals who have detectable calcification. Reassessments of participants in this study after three years will allow associations with the progression of calcification to be assessed.

We found that the presence of CAC was associated with greater blood pressure, lower HDL-cholesterol levels, smoking and greater adiposity. Similar effects have been observed previously [12], [26]. The psychosocial factors analyzed in this study were selected because they have previously been related to coronary heart disease incidence or mortality [27], [28]. As expected, lower SES was associated with less job control, greater financial strain, smaller social networks, greater hostility and lower optimism. The absence of associations between CAC and psychosocial factors is consistent with findings from the MESA study [29], and the Prospective Army Coronary Calcification Study [30], although Kop et al [25] reported that CAC was greater among men and women with smaller social networks.

The pathways responsible for the association between lower grade of employment and more severe CAC are not certain. The relationship was not dependent on differences in lifestyle, biological or psychosocial risk factors. However, other unmeasured factors in these domains may have contributed [16]. Additionally, processes such as disturbed stress responsivity may be relevant. Lower SES is associated with impaired post-stress recovery in cardiovascular and hemostatic responses [31], and with heightened inflammatory cytokine stress responses that may stimulate more rapid progression of coronary atherosclerosis [32].

This study was designed to investigate associations between grade of employment and subclinical coronary artery disease in a healthy sample of older men and women, so we excluded individuals with manifest coronary heart disease or clinically defined conditions such as hypertension and diabetes. It is notable that smoking levels were low, and did not exceed 7% even in the lower SES group, and risk profiles were healthier than in the wider Whitehall II study [16]. Results may not therefore generalize to less healthy sectors of the population. The study was limited to white European participants and was cross-sectional, so causal conclusions cannot be drawn. It was also smaller than other recent studies of SES and subclinical coronary artery disease [10], [11]. The strength of the study is that it was carried out in a cohort in which the index of SES (grade of employment) has already been established as a risk factor for clinical coronary heart disease in prospective longitudinal analyses [16]. The results indicate that lower grade of employment is related to more advanced subclinical coronary artery disease as indexed by arterial calcification independently of lifestyle, biological and psychosocial risk factors. The fact that this relationship was not observed when the presence or absence of CAC was analyzed suggests that lower grade of employment may be particularly relevant once subclinical disease has developed sufficiently to stimulate calcification. Since coronary heart disease risk is markedly increased among individuals with greater calcification [26], our findings reinforce the relevance of socioeconomic disparities to coronary artery disease development.
